# Genetic Control of Muscle Diversification and Homeostasis: Insights from *Drosophila*

**DOI:** 10.3390/cells9061543

**Published:** 2020-06-25

**Authors:** Preethi Poovathumkadavil, Krzysztof Jagla

**Affiliations:** Institute of Genetics Reproduction and Development, iGReD, INSERM U1103, CNRS UMR6293, University of Clermont Auvergne, 28 Place Henri Dunant, 63000 Clermont-Ferrand, France; christophe.jagla@uca.fr

**Keywords:** *Drosophila*, muscle, genetic control, muscle diversification, muscle homeostasis

## Abstract

In the fruit fly, *Drosophila melanogaster*, the larval somatic muscles or the adult thoracic flight and leg muscles are the major voluntary locomotory organs. They share several developmental and structural similarities with vertebrate skeletal muscles. To ensure appropriate activity levels for their functions such as hatching in the embryo, crawling in the larva, and jumping and flying in adult flies all muscle components need to be maintained in a functionally stable or homeostatic state despite constant strain. This requires that the muscles develop in a coordinated manner with appropriate connections to other cell types they communicate with. Various signaling pathways as well as extrinsic and intrinsic factors are known to play a role during *Drosophila* muscle development, diversification, and homeostasis. In this review, we discuss genetic control mechanisms of muscle contraction, development, and homeostasis with particular emphasis on the contractile unit of the muscle, the sarcomere.

## 1. Introduction

### 1.1. General Overview

*Drosophila melanogaster*, a holometabolic insect with a short lifespan, has served as a simple model to study myogenesis [1,2] and contractile proteins [3] for decades. Myogenesis in *Drosophila* occurs in two waves, one during the embryonic stage that gives rise to the larval body wall or somatic muscles and the second during pupal development that gives rise to adult flight, leg, and abdominal muscles [4]. All these muscles are voluntary, syncytial (multinucleate), and striated making them similar to vertebrate skeletal muscles [5]. Multiple signaling pathways, genes, and processes are conserved from *Drosophila* to vertebrates [6,7]. Muscles provide force to ensure various locomotory behaviors such as crawling, walking, jumping, and flying in *Drosophila*. Thus, they need to carry high levels of a mechanical load and are subject to constant strains, which can potentially disrupt homeostasis. Muscle movements need to be precise and coordinated, where communication with other tissues such as the nervous system provides critical inputs [8]. Muscles are the major reservoir for amino acids in the body that contribute to muscle mass and protein homeostasis [9]. All muscle functionalities require that they are correctly formed in the first place to attain a homeostatic state in which they are physiologically active and stable. Muscle intrinsic signaling as well as signaling from external organs contribute to muscle homeostasis. Muscles display a high degree of plasticity or flexibility at the signaling, metabolic, myonuclear, mitochondrial, and stem cell levels.

This review is divided into three parts. The first part presents an overview of the mechanisms of muscle contraction in *Drosophila*. The second part focuses on the development of the larval and adult muscles. In the third part, we discuss the maintenance of muscle homeostasis in normal conditions and the adverse effects of the loss of this homeostasis in pathological conditions. Throughout the review, the focus is on sarcomeres, which are the basic contractile units of the muscle.

### 1.2. Major Structural Components of the Drosophila Muscle and Their Vertebrate Counterparts

In *Drosophila*, muscle function is coordinated by sensory, excitatory, and mechanical inputs by its connection to the nervous system via neuromuscular junctions and to the epidermis via myotendinous junctions akin to vertebrate systems though they present differences, some of which are outlined below.

#### 1.2.1. Sarcomeres

**Figure 1:**
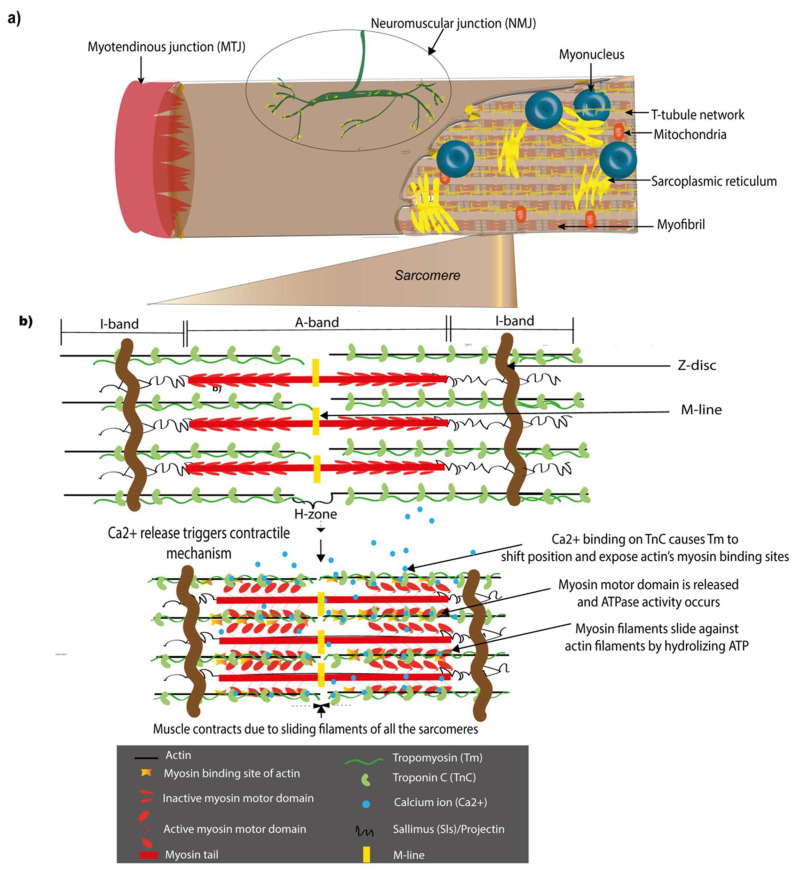
Schematic representation of the larval body wall or somatic muscle structure and the sliding filament theory of muscle contraction. (**a**) Muscle structure with myofibrils and the network of myonuclei, sarcoplasmic reticulum (SR), T-tubules, and mitochondria. The muscle is connected to the nervous system via the neuromuscular junction (NMJ) and to the epidermis via the myotendinous junction (MTJ). Myofibrils are formed of repetitive contractile units, the sarcomeres. (**b**) The structure of a sarcomere and the mechanism of contraction proposed by the sliding filament theory. Ca^2+^ ions released upon neurotransmitter signaling from the NMJ launch a cascade by binding to TroponinC (TnC) on the thin filaments of sarcomeres. This Ca^2+^ binding causes a conformation change in Tropomyosin (Tm) bound to actin, exposing actin’s myosin binding sites. This permits the activated myosin motor domain to bind to actin and slide against it by utilizing the energy stored in Adenosine Triphosphate (ATP).

Sarcomeres are the basic contractile units of the muscle and provide the force for contraction during movements (Figure 1). They are repetitively arranged in a regular pattern that gives a striated appearance under the microscope to vertebrate skeletal muscles as well as *Drosophila* somatic, flight, and leg muscles [10,11]. Sarcomeric length, functional domains, and many component proteins are conserved between invertebrates and vertebrates, although studies also point to interesting differences among species, which appear to be adaptations to individual muscle function [12,13,14,15]. Despite structural differences in *Drosophila* sarcomeric proteins in comparison to vertebrate counterparts, they have similar functional interactions and possess conserved functional domains; for example, the PEVK domain of the *Drosophila* titin, Sallimus (Sls) confers elasticity similar to vertebrates [16]. Thus, the sarcomere provides an example of nature reusing and repurposing components across evolution.

#### 1.2.2. Myotendinous Junctions (MTJs)

In *Drosophila*, the MTJ is an attachment formed between the muscle and specialized groups of tendon-like cells of ectodermal origin called tendon cells, also known as apodemes (Figure 1a). Unlike vertebrates, *Drosophila* does not have an internal skeleton and tendon cells help anchor the muscles firmly to the cuticular exoskeleton instead, which helps transmit the contractile forces to the body to generate motion. This makes them functionally similar to vertebrate tendons despite their distinct embryological origins, mesodermal for vertebrates and ectodermal for *Drosophila* [17,18]. The formation and maintenance of the MTJ is mediated through the ECM by specific integrin heterodimers on the muscle and tendon ends in *Drosophila* similar to vertebrates [19,20,21,22].

#### 1.2.3. Neuromuscular Junctions (NMJs)

The NMJ is the point of contact between the motor neurons of the nervous system and the muscle, which enables environmental inputs to be transmitted via synapses to the muscle (Figure 1a). The *Drosophila* larval NMJ is an established model for NMJ formation and function. This NMJ is glutamatergic and responds to the neurotransmitter glutamate unlike vertebrate NMJs that are cholinergic and respond to acetylcholine. However, they are of particular interest owing to their similarity to mammalian brain glutamatergic synapses that express multiple genes orthologous to *Drosophila* genes and the ease with which NMJ assembly can be studied in this model [23,24,25]. It continues to be an active field of study with focus equally shifting to adult motor neurons formed after metamorphosis [26,27].

## 2. The Sarcomere and Molecular Mechanisms of Muscle Contraction

Voluntary muscle contraction is a highly coordinated process that depends on cooperative signaling from sensory neurons via interneurons and motor neurons to the NMJ of the muscle [28,29,30]. Given that the principal muscle function is to generate movements by contracting, the sarcomeric contractile units are indispensable for muscle function and their maintenance is crucial. The *Drosophila* adult indirect flight muscle (IFM) is established as a model to study sarcomere assembly and the functions of its components [31]. IFMs are built of multiple myofibers and have a stereotypic pattern of sarcomeric proteins forming highly ordered myofibrils similar to human skeletal muscles allowing the study of sarcomere malformations under mutant conditions. The IFM is also a model to study stretch activation (SA) [32]. During SA, there is a high frequency of contraction although the nervous system input frequency is much lower. This is possible due to the delayed increase in tension following muscle stretching. SA is a mechanism found in all muscles though it has particular significance in certain muscle types with rhythmic activity such as human cardiac muscles and the fruit fly flight muscles. In contrast to the multi-fiber IFM muscles of the adult, the somatic muscles in the *Drosophila* embryo and larvae are built of only one muscle fiber per muscle and present a much simpler model to study myofibers.

A sarcomere is a specialized structure adapted for muscle contraction (Figure 1). During myofibrillogenesis, newly formed sarcomeres align in repeating units along the length of a muscle to form a myofibril and multiple myofibrils covered by the plasma membrane form a myofiber. A sarcomere is built of thin-actin and thick-myosin filaments with associated proteins facilitating contraction-relaxation cycles. The thick filaments consist of myosin polymers with each myosin consisting of a myosin tail and two myosin heads, which are capable of attaching to actin during muscle contraction. The two ends of a sarcomere are demarcated by a Z-disc, a huge protein complex that anchors the thin filaments that form I-bands on either side of a sarcomere, while the thick filaments form an A-band in the center (Figure 1). In between the two I-bands is an H-zone lacking myosin heads and in the center of the H-zone is an M-line that corresponds to another large protein complex that anchors the thick filaments [33].

Sarcomere function is intricately linked to other organelles such as the mitochondria [34], myonuclei [35], sarcoplasmic reticulum (SR), and T-tubules [10,36]. The efficient function of sarcomeres is closely coupled with the periodic arrangement of the SR and T-tubules around them [10,36,37,38]. T-tubules are regular tubular invaginations of the plasma membrane at each sarcomere. The membrane organelle SR is linked to the myonuclei and T-tubules to facilitate the exchange of proteins and ions. The SR is the major intracellular reservoir of calcium (Ca^2+^) ions in the muscle, which are essential for muscle contraction. The T-tubule and SR form a specialized triad/dyad structure, which is indispensable for correct muscle functioning by excitation-contraction (EC) coupling. This EC coupling enables the transmission of excitation potentials from the NMJ to the SR, which triggers Ca^2+^ release from the SR that in turn initiates sarcomeric sliding movements leading to muscle contraction. Apart from Ca^2+^, other ions contribute to muscle contraction [39]. The Na^+^K^+^-ATPase is a Na^+^-K^+^ pump that can pump Na^+^ out of and K^+^ into the cells against their normal concentration gradients. In muscles, the concentration of these ions fine-tunes the force of contraction [40]. In *Drosophila*, muscles are one of the major organs that express the Na^+^K^+^-ATPase α subunit [41]. One form of the Na^+^K^+^-ATPase β subunit, Nrv1 interacts with Dystroglycan (Dg), which is part of a complex that helps transmit forces into the muscle cell [42].

The mechanism of muscle contraction is explained by the sliding filament theory [43,44], reviewed by Hugh Huxley [33]. This theory proposes that the myosin head domain acts as a motor and slides against the actin filament powered by the energy stored in ATP. This sliding of the central myosin along the thin filaments causes the two I bands on either side to come closer to each other. During contraction, environmental inputs are transmitted by the nervous system to the NMJ leading to Ca^2+^ binding to the Troponin C (TnC) subunit of the Troponin (Tn) complex. This leads to the Troponin T (TnT) subunit that binds to the actin binding protein Tropomyosin (Tm) triggering a conformational change in Tm, thus shifting its position on actin and exposing the myosin binding site of actin [45,46,47]. Myosin that is turned ‘on’ by a myosin regulatory light chain (Rlc) phosphorylation [48] liberates the motor domains in the myosin head that were folded onto the myosin tail, thus facilitating its binding to actin. Subsequent ATP hydrolysis and energy release, thanks to its ATPase activity, permits it to move along the thin filament to contract the muscle. For the muscle to relax, the Troponin I (TnI) troponin subunit inhibits the actomyosin interaction [49] so that Tm covers the myosin binding site of actin and the myosin is switched ‘off’ and folded back onto the myosin tail [50,51]. This coordinated key muscle function highlights the importance of ionic and sarcomeric component homeostasis in muscles, which implies the supply and maintenance of the right quantities of the right ions and sarcomeric components at the right time to ensure muscle functionality.

During contraction, the MTJ helps anchor the myofibrils and transmits forces [19,52]. Tight interactions between sarcomeric components ensure myofibrillar integrity and prevent disintegration due to contractile forces. CapZ binds to the actin barbed end and links it to the Z-disc [13] while Z-disc proteins such as the filamin Cher [53], Zasp, and α-actinin anchor the thin filaments [54]. Similarly, the M-line protein Obscurin that associates with the thick filament [55], Muscle LIM protein at 84B (Mlp84B) that cooperates with Sallimus (Sls) known as the *Drosophila* titin [56], integrins [57], and other proteins ensure muscle integrity. Sarcomeres are subject to constant mechanical strain due to the thin and thick filament friction and need to be consistently replenished to ensure their function over a lifetime. Since these muscles are voluntary, they also need to be able to stop contracting at will and go back to their natural state. Defective sarcomeric formation, maintenance, and homeostasis are associated with muscular diseases [15,58].

## 3. Muscle Diversification—On the Road to Muscle Homeostasis

Muscle development is a finely orchestrated, synchronized process that occurs in spatial and temporal coordination with the development of other communicating tissues to finally form a homeostatic muscle. There are similarities as well as differences between *Drosophila* and vertebrate myogenesis [59]. During development, each muscle diversifies to attain an identity tailored to its specific functional requirements. The study of muscle diversification during development is of interest in the context of homeostasis for two primary reasons:

(a) Events similar to those occurring during development need to be reinitiated to repair and regenerate an injured muscle and reestablish muscle homeostasis [60]. This is a new field of study in *Drosophila* stemming from the recent discovery of muscle satellite cells in adult flies [61].
(b) The two waves of myogenesis in *Drosophila* result in two homeostatic states, one in the larva and one in the adult. The larval homeostatic states are highly dynamic given the large growth spurt that occurs over the three larval instars. This might provide insights into mechanisms of muscle atrophy and hypertrophy. Forkhead box sub-group O (Foxo), for example, has been shown to inhibit larval muscle growth by repressing *diminutive* (*myc*) [62]. In mice, excess c-Myc has been shown to induce cardiac hypertrophy [63].

### 3.1. Embryonic Myogenesis of Larval Muscles

Embryonic myogenesis gives rise to monofiber larval somatic muscles whose main function is to aid in hatching and the peristaltic, crawling movements of the larvae. The embryonic and larval somatic musculature consists of a stereotypical pattern of muscles in each segment, with 30 muscles in most abdominal hemisegments (A2–A6) (figure in Table 1). There are fewer muscles in the posterior and first abdominal hemisegment and a slightly different set of muscles in the three thoracic hemisegments (T1–T3). Embryonic muscles arise from the mesoderm germ layer and their development requires intrinsic mesodermal cues and extrinsic cues from the adjacent epidermal and neural cells. Thus, they develop in synchrony with the development of muscle-interactors such as tendon cells and motor neurons and need to ‘speak a common language’ to communicate for coordinated development and maintenance.

Somatic muscle specification and differentiation have been reviewed extensively in the past [1,2,7,60,61,62] and this review presents complementary as well as new information that emphasizes the role of developmental factors in future muscle homeostasis.

#### 3.1.1. Muscle Diversification by the Specification of Muscle Founder Cells Expressing Identity Transcription Factors (iTFs)

The embryo undergoes gastrulation by invagination [64], which brings the three germ layers, the ectoderm, the somatic muscle forming mesoderm, and endoderm in juxtaposition with each other. This helps provide extrinsic signals to the developing mesoderm. Following this juxtaposition, the mesoderm is divided into domains by morphogenic signaling [65] giving rise to a somatic muscle domain in which the transcription factor (TF) Twist (Twi) provides a myogenic switch [66]. Subsequently, equivalence or promuscular cell clusters expressing the neurogenic gene *lethal of scute* (*l’sc*) form and one muscle progenitor cell is singled out from each cluster by lateral inhibition involving Notch and Ras/MAPK signaling [67,68]. The remaining Notch activated cells in the equivalence groups become fusion competent myoblasts (FCMs). This process is reminiscent and coincides temporally with the specification of neural lineages from the neurectoderm [69,70], which occurs during embryonic stages 8–11, while muscle cell identity specification occurs during stages 9–11.

**Figure 2:**
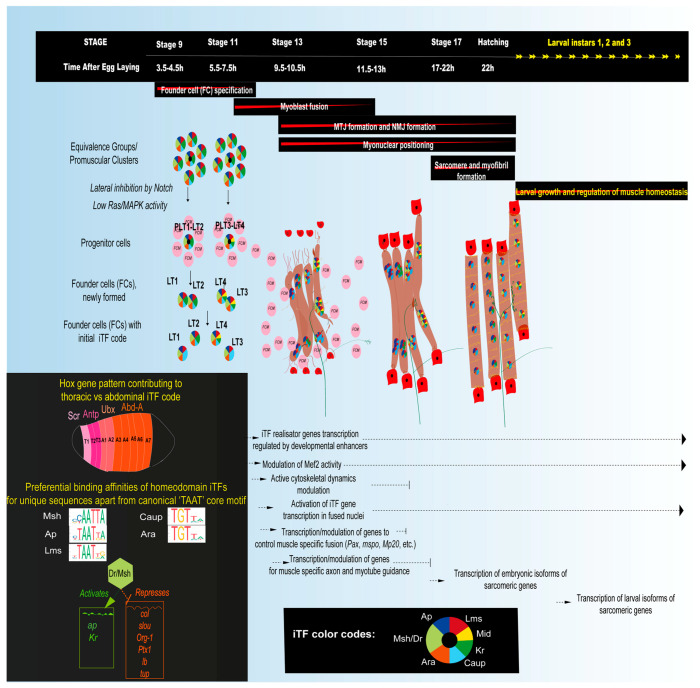
Spatial and temporal expression muscle identity transcription factors (iTFs) of the larval lateral transverse (LT) muscles. Sizes are not up to scale. Following the specification of progenitor cells by a lateral inhibition by Notch and low Ras/MAPK activity, founder cells (FCs) expressing muscle specific iTFs are specified for each LT muscle, LT1, LT2, LT3, and LT4 with a contribution from homeobox (Hox) genes to specify thoracic versus abdominal identities. Each iTF has preferential binding abilities to certain enhancers. The iTF expression is followed by the regulation of transcription and modulation of expression of their realisator genes which establish muscle identity over the course of development. The spatial and temporal expression of iTFs coupled with their modulation of realisator genes, which include generic muscle genes, in collaboration with Mef2 begs the question about their contribution to muscle homeostasis. Abbreviations: FCM: Fusion competent myoblasts; FC: Founder cells; LT: Lateral transverse muscles; iTF: Identity transcription factor.

The singled-out muscle progenitors divide asymmetrically to give rise to founder cells (FCs), which are believed to carry all the information necessary to give rise to the diversity of muscle types. Asymmetric divisions of progenitors can give rise to two FCs, an FC and a Numb negative adult muscle precursor (AMP) or an FC and a cardiac progenitor, which subsequently migrate away from each other [67,71,72]. Each FC contains the information to establish one muscle’s identity since it can form correct attachments and be correctly innervated even in the absence of myoblast fusion with surrounding FCMs [73,74]. It expresses its characteristic code of TFs known as muscle identity transcription factors (iTFs) (Figure 2). The expression of a combinatorial code of iTFs in distinct progenitors is the result of their spatial positioning as well as tissue specific convergence of multiple signaling cascades [75]. For example, Wg signaling from the adjacent developing central nervous system (CNS) is implicated in the specification of Slouch (Slou) positive FCs [76] highlighting the importance of coordinated tissue development.

#### 3.1.2. The Role of iTFs

After the initial discovery of distinct Slou expressing FCs [77], many other TFs expressed in discrete subsets of FCs were subsequently identified and collectively named muscle identity transcription factors or iTFs (Table 1). A loss or gain of iTF function can cause muscle loss [78,79] or transformation of one muscle to another muscle fate [80,81] and impede muscle development [82] thus disrupting muscle patterns. The iTFs such as Ap, Slou, Eve, Kr, Lb, and Lms are also expressed in the CNS [78]. Many identified iTFs such as Dr/Msh, Lms, Ap, Ara, Caup, Lb, Slou, Eve, Ptx1, and Tup are homeodomain TFs that are known to recognize similar canonical TAAT containing binding motifs, but they could have preferential high affinity binding motifs (Figure 2), as has been shown for Slou [83] and Caup [84]. The iTFs from other TF families are Twi, Nau, Kr, Kn/Col, Mid, Six4, Poxm, Org-1, and Vg. Newly identified iTFs for a subset of dorsal muscles are Sine occulis (So), No ocelli (Noc), and the cofactor ETS-domain lacking (Edl) [85], which act sequentially with their cofactors. The iTF Vg also acts with a cofactor, Sd [86].

The iTF code can be hierarchic and activate other iTFs as has been shown for Org-1 that activates Slou and Lb [98]. In addition to hierarchy, there seems to be isoform specificity in iTF expression [85]. Certain iTFs confer identity by repressing other iTFs. Dr, for example, represses Lb that is normally active only in the SBM muscle and Eve that is normally continually expressed in only the DA1 muscle [104], while Tup represses Col in DA2 [81]. The expression levels of one isoform of the chromatin remodeling factor Sin3A is implicated in modulating the response to iTFs by acting on the *slou* enhancer [105].

The same iTFs can be expressed in different muscles, but with different co-iTFs. The identity code for one specific muscle subset, the lateral transverse or LT muscles, which comprises the four muscles LT1-4, for example, is known to be set up by a combinatorial expression of Dr [89], Ap [78], Kr [93], Lms [95], and the Ara/Caup complex [87] (Figure 2). Dr appears to directly or indirectly activate the transcription of many LT iTFs such as *Kr*, *ap*, and itself while repressing non-LT iTFs such as *col*, *slou*, *Org-1*, *Ptx1*, *lb*, and *tup* [83] and its expression is lost by mid embryonic stages. Only Lms is specific to all four LT muscles while others are also expressed in other muscle subsets, although not in combination with the same co-iTFs. This seems to be the way the iTF code is set up, where they are repurposed in different combinations to define the identity of different muscles [81,85,88]. Even amongst the LT muscles, each muscle has a specific combination of these iTFs (Figure 2). Some iTFs such as Lms are persistently expressed while others such as Ap and Kr are transient. A characteristic feature of Kr is that it is transiently expressed and subsequently lost from one of the two sibling FCs that arise from the progenitor that expresses it [87,93].

The thoracic LT muscles have slightly different characteristics such as a different number of myonuclei, which might depend on the iTF code along with individual iTF dynamics in conjunction with other TFs [106]. Homeotic or Hox genes such as *Antennapedia* (*Antp*), *Abdominal-A* (*Abd-A*), *Abdominal-B* (*Abd-B*), and *Ultrabithorax* (*Ubx*) control the muscle pattern along the anterior-posterior axis and are thus part of the iTF code [106,107,108] (Figure 2). The mechanism of Hox gene regulation in muscles could be by repressing genes specifying alternative fates by altering the epigenetic landscape in a tissue specific manner [109]. Hox genes could also be involved in the coordination of the proper innervation of muscles [110].

Once an FC initiates its diversification with a specific identity determined by an iTF code, it starts differentiating by activating realisator genes acting downstream of iTFs. Some muscle identity realisator genes have been identified. They include several muscular differentiation genes such as *sallimus* (*sls*), *Paxillin* (*Pax*), *Muscle protein 20* (*Mp20*), and *M-spondin* (*mspo*), which are differentially expressed in muscle subsets to control the acquisition of specific muscle properties such as the number of myoblast fusion events or the specific attachment to tendon cells. [111,112]. Thus, an iTF code and a downstream realisator gene code are both essential to generate a diversity of muscle types with specific functions and to set the foundation for muscle homeostasis.

#### 3.1.3. Mef2, a Key Muscle Differentiation Factor and Its Interactions with iTFs

The *Drosophila* Myocyte enhancer factor 2 (Mef2) acts along with iTFs and their realisator genes to cause the muscle to differentiate. Mef2, similar to its vertebrate ortholog MEF2, is indispensable for muscle differentiation [113,114,115]. It has an equally important role in fully differentiated muscles and the control of its expression and activity is dynamic. Though it is expressed in the mesoderm during all stages, its loss of function does not prevent initial muscle specification and FC generation, but completely blocks subsequent differentiation so that muscle cells undergo apoptosis in later embryonic stages [116,117]. Mef2 activity levels change over time and appear to be adapted to varying target gene expression requirements during different developmental stages [86,118]. It regulates a vast array of muscle specific genes [119,120], sometimes in cooperation with other TFs such as Cf2 [121,122]. It is itself regulated by various mechanisms including autoregulation [123], signal and TF integration at its specific cis regulatory modules (CRMs) [124], or post transcriptionally by highly conserved miRNAs such as miR-92b [125]. TFs such as Twi and Lameduck (Lmd) [119,126] acting on muscle specific CRMs and Akirin-bearing chromatin remodeling complexes [127] are known regulators of Mef2 transcriptional activity. The RNA modifying enzyme Ten-eleven-translocation family protein (Tet) shows a strong overlap with Mef2 expression in somatic muscles and its depletion in muscle precursors leads to larval locomotion defects [128] though the relationship between the two factors is unclear.

The iTFs Vg and Sd physically interact with each other and with Mef2 either alone or in combination [129]. Each of them has a spatially and temporally controlled expression pattern and altering their expression levels severely affects the development of specific ventral muscles during late stages by affecting the levels of realisator genes. Thus, iTFs could play a key role in the modulation of Mef2 interactions. Given the central role of Mef2 in muscle development, disrupted Mef2 expression can have deleterious consequences at all stages of muscle development and maintenance.

#### 3.1.4. Myoblast Fusion and Myonuclear Positioning

In order to form a differentiated muscle, in the mid-stage embryo, a specific number of neighboring FCMs fuse with the FC to form a syncytium (Figure 2). The formation of syncytial fibers by myoblast fusion is complete by the end of stage 15 [130,131,132]. As fusion proceeds, the round-shaped FC becomes a myotube that elongates, becomes polarized, and locally sends out filopodia in the presumptive area of MTJ and NMJ formation. Fusion involves complementary cell adhesion molecules (CAMs) such as Dumbfounded (Duf) or its paralogue Roughest (Rst) expressed on FCs [133,134] and Stick and Stones (Sns) or its paralogue Hibris (Hbs) expressed on FCMs [135,136], respectively. They trigger a signaling cascade, thereby modulating cytoskeleton dynamics to form a fusogenic synapse that helps integrate the FCM nucleus into the FC/myotube. In *Drosophila*, the iTF code dictates the number of fusion events by controlling the expression level of fusion genes encoding actin cytoskeleton modulators such as *Muscle Protein 20* (*Mp20*) and *Paxillin* (*Pax*) or the ECM component *m-spondin* (*mspo*) [112]. A recent study provides insights into the FCM-FC transcription dynamics in a syncytial myotube [137]. FCMs appear to be naïve and respond to the local environment that recruits them for fusion. Upon fusion, the FCM adopts an FC transcriptional program triggered by the transcription of certain muscle specific iTFs. However, once fusion is complete, differences in gene transcription among myonuclei within the same muscle are observed. For example, not all myonuclei transcribe the iTFs at a given timepoint, which could help maintain an mRNA-protein balance. Evidence from this study suggests that even after fusion is complete the FC nucleus that seeded the muscle retains a transcriptional program that is distinct from other myonuclei.

As fusion proceeds, at around stage 14, the nuclei of newly fused FCMs start exhibiting characteristic movements until they are positioned peripherally to maximize the internuclear distances. This process, also observed in vertebrate muscles [138,139], has been extensively studied in the LT muscles in the *Drosophila* embryo. In these muscles the new myonuclei initially cluster into two groups, unlike in vertebrates where nuclei cluster in the center of the myotube [140], then disperse and are finally arranged along the periphery of the myotube. Correct myonuclear positioning is dependent on the LINC complex [141] that links the inner nuclear membrane (INM) to the outer nuclear membrane (ONM) and the ONM to the microtubules (MT) and the actin cytoskeleton. Mispositioned myonuclei in *Drosophila* larvae cause locomotion defects, and in humans, are associated with various diseases [142]. This is not surprising considering the close association of myonuclei with muscle structural components such as the NMJ, MTJ, actin cytoskeleton, microtubule, SR, Golgi complex, and T-tubules. During the larval growth spurt following hatching, myonuclei increase in size along with the increasing muscle size by Myc dependent endoreplication to adapt transcription to muscle functionality requirements [62].

#### 3.1.5. Myotendinous Junction (MTJ) Formation

MTJ formation has been previously reviewed [17,143,144,145]. Once FCs are specified, they migrate towards the ectoderm while tendon precursor cells are specified in the ectoderm in parallel in a muscle independent fashion by the induction of expression of the early growth response factor (Egr)-like zinc finger TF, Stripe (Sr). Interestingly, tendon progenitor cells in mice express the Sr orthologs, the early growth response TFs EGR1 and EGR2 [146]. The StripeB (SrB) isoform is induced during the precursor stage to maintain the tendon cells in a non-differentiated state until later when they differentiate following signals from the approaching muscles. These signals lead to an increase in the expression of the StripeA (SrA) isoform in an integrin dependent manner by promoting *stripe* splicing by the short isoform How(S) of the splice factor interactor How [147]. SrA induces the expression of tendon differentiation markers such as *short stop* (*shot*), *delilah* (*dei*), and *β1-tubulin* (*β1-tub*). At stage 14, tendon cells guide myotubes to their final attachment sites. The targeting of muscles to tendon cells at stage 15 is facilitated by muscle type dependent and generic CAMs as well as signaling molecules. These include Slit-Robo [148] in some ventral muscles, Derailed (Drl) [149] in LT muscles, Kon-tiki (Kon), Glutamate receptor interacting protein (Grip), and Echinoid (Ed), probably involving integrin complexes [149,150,151,152]. Once muscles target their tendon cells, integrin complexes assemble on the muscle and tendon cells facilitated by the αPS2-βPS integrin heterodimer on the muscle end and αPS1-βPS on the tendon cell end to stabilize the attachments [153]. Each attachment site is muscle type specific and the iTF code could potentially modulate the expression of genes such as *kon* [137].

MTJ formation is complete by the end of stage 16 and is then further refined to withstand contractile forces. Talin phosphorylation contributes to MTJ refinement [154]. This is followed by myofibril maturation and attachment to the MTJ. Once muscles start contraction, mechanical forces stabilize the MTJ by reducing integrin turnover [155]. The MTJ grows along with the massive larval growth spurt following hatching.

#### 3.1.6. Sarcomere Assembly and Myofibrillogenesis

Sarcomere assembly has been extensively studied in the *Drosophila* indirect flight muscles (IFM) [31] and other invertebrate models as well as in vertebrate models and cultured human cells [156,157]. These studies point to similarities as well as differences in vertebrate and insect muscles. The premyofibril theory that is widely accepted for vertebrate sarcomere assembly proposes the formation of premyofibrils along the cell periphery containing non muscle myosin, which then incorporate muscle myosin to form nascent myofibrils that subsequently form mature myofibrils [158,159]. In the early stages, distinct Mhc positive fibrils and I-Z-I complexes containing thin filaments protruding from α-actinin positive central Z bodies are seen in invertebrates as well as vertebrates [160,161]. In *Drosophila*, it has been proposed that the individual components of the sarcomere are assembled separately as latent complexes and are then assembled into sarcomeres without assembling into premyofibrils [162,163,164]. Most studies on *Drosophila* sarcomere assembly have been using the IFM as a model and not much attention has been given to embryonic sarcomere development.

In the *Drosophila* embryo, sarcomere assembly is initiated at stage 17. Individual sarcomere constituents are first assembled and then integrated into a mature sarcomere by integrin dependent interdigitation [162]. The precise stage at which each sarcomere component is added is currently not known. Certain sarcomeric proteins such as actin [165] and myosin [166] express sarcomere specific as well as generic cytoplasmic isoforms with roles in other muscle components such as the MTJ. TFs such as Mef2, Chorion factor 2 (Cf2), and E2F transcription factor 1 (E2f1) have been shown to regulate the expression of sarcomeric genes [122,167]. *Drosophila* has six actin isoforms including Act57B and Act87E that are muscle specific and incorporate into larval sarcomeres [165]. Thin filament formation and elongation requires actin binding factors such as the *Drosophila* formin Dishevelled Associated Activator of Morphogenesis (DAAM) [168] and Sarcomere length short (Sals) [169], which localize to the growing thin filament pointed ends. Once the thin actin filament attains its final length, it is capped by a short embryonic isoform of Tmod [169,170]. While non-muscle myosin is a component of premyofibrils in vertebrates, this does not seem to be the case in *Drosophila*, which has only one non-muscle myosin, Zipper (Zip). During stage 16, it colocalizes with PS2 integrin at muscle attachment sites and at stage 17 when sarcomeres form, it also colocalizes to Z-discs and is essential for myofibril formation [162,171]. PS2 integrin follows a similar expression pattern in culture with initial occurrence at contact sites, then at Z-discs [172]. The observation of Zip association with PS2 before sarcomere assembly is significant because myofibrils attach to the MTJ via integrin complexes with Zip acting downstream of PS2 signaling [52]. Therefore, it would appear that the embryo is getting individual components ready for future integration into myofibrils.

By stage 17 of embryogenesis, several sarcomere proteins localize to Z-discs and thin and thick filament organization and myofibril structures are seen. A knockdown of Z-disc proteins Zip, Zasp, and α-actinin at this stage disrupts sarcomerogenesis [162], though Zasp mutant sarcomeres disintegrate after initial correct formation. The myoblast fusion protein Rolling pebbles (Rols7) also colocalizes to the Z-discs during sarcomerogenesis [173], but its function remains to be elucidated. Integrins are distributed along the width of the muscle and align with Z-discs during embryonic sarcomerogenesis. Their loss results in clumping, where I-Z-I body components stay distinct from Mhc containing components. In addition, integrins associate with the ECM and mutant larvae for the ECM type IV collagen Col4a1 present abnormalities in thin-thick filament interdigitation and the degeneration of body wall muscles [162,172,174]. They are also present at epidermal muscle attachment sites along with several Z-disc proteins. Mature sarcomeres align themselves to form myofibrils that attach to the MTJ via the terminal Z-disc to be able to sustain muscle contractions [162,173].

Auld and Folker showed that myonuclear movements are intricately linked to sarcomere and myofibril formation [35]. Their study showed that the Z-disc protein Zasp66, one of the *Drosophila* Zasp family of proteins, localizes as puncta to the cytoplasmic face of the nuclei along with F-actin during initial stages of sarcomerogenesis. At later stages, puncta were observed throughout the muscle. They showed that LINC complex components such as Klarsicht (Klar) and Klaroid (Koi) coordinate initial colocalization of puncta around the nucleus. However, Z-disc-like structures still formed and aligned into myofibrils in LINC component depleted muscles, although they had altered morphology suggesting a specialized role for myonuclei-associated Zasp66 puncta. *sals* mutants display clustered myonuclei at muscle ends as well as myofibrils with numerous shorter sarcomeres suggesting a role for correct myonuclear positioning in myofibril organization [169].

Embryonic myofibrillogenesis within the egg is complete by late stage 17. Asynchronous, episodic contractions occur during the process of myofibril assembly, but coordinated contractions only occur later after mature NMJ formation results in adequate motor inputs [175,176]. Following hatching, during larval stages when the muscles rapidly grow in size, new sarcomeres are generated and organized into myofibrils during an approximately five-day period [62]. In fully mature larval muscles, T-tubules and the SR organize themselves around each sarcomere for excitation-contraction coupling and this organization is Amphiphysin (Amph) dependent [37]. The iTF code could play a role in modulating the muscle specific expression of sarcomeric genes, as has been shown for Vg and Sd that form a complex with Mef2 to modulate Mef2 targets involved in sarcomerogenesis including *Act57B* and *Mhc* [129].

#### 3.1.7. Innervation and Neuromuscular Junction (NMJ) Formation

The development of the NMJ of larval somatic muscles has been previously reviewed [24,25,177,178,179] and represents another example of intricate communication between two different tissues. After neuroblasts differentiate into motor neurons (MNs) in parallel with FC specification [180,181,182], their dendrites in the CNS are organized in a ‘myotopic map’ reflecting the innervation pattern of their target muscles and MNs can reach target locations even in the absence of muscles [182]. Each neuroblast expresses a characteristic code of TFs that defines its identity as is the case for muscle FCs expressing iTFs [26]. By stage 12, MN axons fasciculate in each hemisegment within three peripheral nerves, the intersegmental nerve (ISN), segmental nerve (SN), and transverse nerve (TN) that extend towards specific target muscles from the ventral nerve chord (VNC). The ISN, SN, and TN branch stereotypically as they extend growth cones towards muscles to form MN nerve branches that further defasciculate into axons to innervate muscles. The SN nerve, for example, branches into SNa, SNb, SNc, and SNd with a subset of MNs from the SNa innervating a muscle subset including LT muscles [181].

At around stage 14, each MN extends numerous filopodia from axon growth cones towards muscles to explore their target muscles. Muscles in turn extend myopodia that cluster together on axon growth cone arrival and intermingle with growth cone filopodia. Muscles also form lamellipodia during innervation [183]. Target muscle recognition and contact are facilitated by muscle and MN specific CAMs, Cell Surface and Secreted (CSS) proteins, and other proteins [184]. Certain guidance molecules such as the homophilic Connectin (Con) are expressed in the SNa MN as well as the LT muscles it innervates [185]. Con is also expressed in the DT1 muscle and its expression is potentially modulated by the iTF code [137]. Some MNs and the muscles they innervate express the same iTF, as is the case for the Eve expressing DA1 muscle and its innervating aCC MN in the ISNb [91,181,182,186]. Eve indirectly modulates the MN expression of the Netrin repulsive presynaptic receptor Unc-5 in the ISNb [187] to guide MN axons. Upon MN contact, muscles start to accumulate Glutamate Receptor (GluR) at synaptic zones mediated by Disks large (Dlg) to form primitive synapses in an innervation dependent fashion [188,189]. By the end of stage 17, non-target synapses are pruned and mature synapses form, which exhibit a stereotyped morphology of boutons with active zones for vesicle release on the presynaptic end and novel synthesis and clustering of more GluR on the postsynaptic end [190]. Once NMJ formation is complete, muscles are ready to contract in a coordinated manner.

During larval stages, some MNs are remodeled and this is reflected in the larval CNS myotopic map [191]. Until the third larval instar, the NMJ grows by arborization and addition of boutons, a process that requires the gene *miles to go* (*mtgo*), which is an ortholog of mammalian *FNDC3* genes [192], and integrins [193]. There is also an activity dependent refinement of the synapse mediated by Ca^2+^ [194]. Tenurins, a conserved family of transmembrane proteins enriched in the vertebrate brain that possesses glutamatergic synapses are implicated in *Drosophila* axon guidance as well as synaptic organization and signaling with muscle specific expression [195].

As muscles form, abdominal adult muscle precursors (AMPs) arrange themselves in niches between specific peripheral nerves and muscles. They form an interconnected network connecting to each other and to the peripheral nerves by extending filopodia [196,197,198]. All embryonic muscle development processes finally lead to the formation of functional larval body wall muscles that closely communicate with the epidermis via the MTJ and with the nervous system via the NMJ to ensure larval locomotion.

### 3.2. Pupal Myogenesis of Adult Muscles

Adult muscles are generated during a second wave of myogenesis during pupal metamorphosis where most larval muscles are histolyzed. Metamorphosis marks the end of larval muscle homeostatic states. Adult flies have a pair of wings in the thoracic segment T2 and three pairs of legs in thoracic segments T1–T3, which are powered by specialized thoracic flight and appendicular muscles, respectively (figure in Table 2). Adult myogenesis has been reviewed recently in [199,200]. All adult thoracic muscles including the indirect flight muscles (IFMs), direct flight muscles (DFMs), and leg muscles possess a multi-fiber structure similar to vertebrate skeletal muscles. However, unlike heterogenous mammalian skeletal muscles with one muscle composed of slow and fast fiber types, each *Drosophila* muscle appears to have a single fiber type. IFMs are constituted of the dorsoventral muscles (DVMs) and the dorsal longitudinal muscles (DLMs), which facilitate upward and downward wing strokes respectively during flight. The muscle fibers that build the IFM and leg muscles differ in organization and morphology to adapt to different functionalities. The IFMs are fibrillar, asynchronous muscles while the tergal depressor of the trochanter (TDT) or leg jump muscles and DFM are tubular, synchronous muscles [201,202,203]. Similar to mammals, individual fiber types in the adult fly differ in component constitution such as expressing specific myosin heavy chain isoforms [203,204]. The generation of adult muscles is initiated by a series of coordinated processes again requiring close communication between tissues.

#### 3.2.1. Myoblast Pool Generation by Adult Muscle Precursors (AMPs) during Larval Stages

Embryonic myogenesis sets the foundation for adult muscle development since the asymmetric divisions of embryonic muscle progenitor cells give rise to adult muscle precursors (AMPs) in addition to the embryonic muscle FCs. AMPs are Notch positive, Numb negative cells that remain quiescent with persistent Twist (Twi) expression until initial pupal stages when they get reactivated [205] and contribute to adult muscle development. Abdominal AMPs are closely associated with the larval muscles and with the peripheral nervous system (PNS) enabling crosstalk and providing positional cues to the AMPs that give rise to adult abdominal muscles [196,197,198]. In the thoracic segments, AMPs associate with wing and leg imaginal discs, which are epidermal cell clusters set aside in the embryo and larva and act as precursors for the future generation of adult wings and legs, respectively [206]. During the first and second instar larval stages, these AMPs undergo symmetric divisions giving rise to an imaginal disc associated monolayer of Twi and Notch positive adepithelial cells. In the abdominal segments, they proliferate while remaining associated with their muscle fibers similar to vertebrate satellite cells [207,208]. During the third larval instar, due to the activation of Wg signaling from the imaginal discs, AMPs undergo asymmetric divisions forming one stem cell and one Numb positive post-mitotic myoblast where Notch signaling is inhibited [209,210]. Thus, a large pool of myoblasts is primed for metamorphosis.

The myoblasts primed to form IFM express high levels of Vestigial (Vg), which represses Notch and promotes IFM differentiation [211], and low levels of the TF Cut (Ct) while DFM myoblasts express high Ct levels, with the levels being governed extrinsically by the ectoderm [212]. DFM myoblasts also express Lms [95]. The myoblasts associated with the leg imaginal disc on the other hand express Ladybird (Lb) similar to vertebrate limb bud myoblasts that express the Lb orthologue LBX1 [213,214], which represses Vg. Mutant *vg*, *ct*, *lms*, and *lb* flies have severely disrupted muscle pattern or function and they thus contribute to the adult muscle iTF code (Table 2). Vg, Lms, and Lb also act as embryonic somatic muscle iTFs expressed in a subset of embryonic muscles [86,94,95] (Table 1). Among embryonic myogenic factors, it was noticed that Apterous (Ap) expression defines all prospective flight muscle epidermal muscle attachment sites in the wing disc [215]. Similar to embryonic stages, Duf positive adult FC specification takes place by the third larval instar, but in contrast to embryos it is driven by Heartless (Htl) mediated Fibroblast growth factor (Fgf) signaling and Hox genes [212,214,216,217].

#### 3.2.2. Histolysis of Larval Muscles, Adult iTF Code Refinement, and the Contribution of AMPs

During pupal stages, most of the larval muscles are histolyzed in the thoracic as well as abdominal hemisegments [221,222]. Myoblasts generated from AMPs either fuse with non-histolyzed larval muscle scaffolds to which they associate or give rise to adult muscles de novo [221]. In the T2 mesothoracic segment, three larval dorsal oblique muscles, DO1, DO2, and DO3 escape histolysis and serve as templates for the formation of the DLMs while the DVMs and leg muscles are generated de novo. At the end of the third larval instar, the myoblasts start expressing the muscle differentiation factor Mef2 in an ecdysone dependent manner [123]. As with embryonic myogenesis, adult muscle formation is seeded by FCs, with the number of FCs generated corresponding to the number of muscles they will seed [217]. The DLMs are an exception where the three remnant larval muscles serve as FCs and express the marker Duf. Nevertheless, if the larval muscles giving rise to adult DLMs are ablated they still form muscles de novo by an innervation dependent process, although with aberrations [223].

During early pupal stages myoblasts start migrating. MNs play a significant role in initial adult myogenesis by regulating myoblast proliferation during the second larval instar and subsequent myoblast migration during pupal stages. In denervated flies, DVM muscle formation is severely compromised and it leads to the reduction in DLM size when using larval muscles as templates whereas if larval templates are ablated, their de novo formation is abolished [223]. In the abdominal segments, myoblasts migrate and associate with nerves to form adult muscles [224]. In the thoracic segments, the wing and leg discs evaginate and myoblasts migrate along them to reach their destinations where adult muscles are generated. The myoblasts either fuse with FCs or with larval templates using a similar machinery to embryonic myoblast fusion to form fully differentiated adult muscles by 36 h after puparium formation (APF). Muscles extend as they fuse and attach to the MTJ on either end [221,225,226].

Apart from Vg, Ct, and Lb that act as adult muscle iTFs (Table 2) to confer myoblast identity in the imaginal discs during larval stages, the expression of the embryonic iTF Ap is initiated during pupal stages in myoblasts that will give rise to the DFM but not IFM in addition to epidermal attachment sites [215]. Unlike the embryonic FCs, it is expressed in adult FCMs instead of adult FCs, but similar to the embryonic FCs they contribute to the same muscle’s iTF code along with Lms. This hints at specific muscle patterning information derived from these iTFs. Ap is necessary for the correct formation of DFMs and continues to be expressed in fully formed DFMs. It is also necessary for IFM attachment by regulating Stripe (Sr) expression which, similar to the embryo, is essential for adult muscle attachment. In *lms* mutants, the wing disc Vg domain is expanded and although muscles seem normal, the adult wings exhibit a held-out phenotype suggesting contraction abnormalities [95]. As fusion begins, the IFM FCs also express the adult iTFs Extradenticle (Exd), Homeothorax (Hth), and Spalt major (Salm), which genetically interact to specify a fibrillar versus tubular fate by regulating fiber specific gene expression and splicing regulated by Arrest (Aret) [201,218,219]. The iTF Erect wing (Ewg) also significantly contributes to IFM identity [220].

#### 3.2.3. MTJ Formation

The wing and leg imaginal discs generate Sr positive tendon-like precursor cell clusters starting from the third larval instar until the beginning of pupation. Sr expression is initiated by Notch signaling [227,228]. Leg muscles attach to the internal tendons on one end and the tendon cells in the exoskeleton on the other end. At about 3 h APF, the leg disc Sr positive tendon precursor cells invaginate into an evaginating leg disc and are closely associated with myoblasts that give rise to leg muscles [18]. Disrupting tendon precursors also disrupts myoblast localization. The epidermal tendon precursor cells’ shape changes to form tubular structures during invagination giving rise to internal tendons to which each leg muscle attaches on one end with tendon specificity. DLM muscles that form by the splitting of remnant larval muscles extend filopodia on either end as they grow and split. Still in the process of splitting, their filopodia interdigitate with those of their target tendon cells and initiate MTJ formation that requires Kon, integrins, Tsp, and Talin similar to embryos. DLM filopodia disappear after a mature MTJ forms by 30 h APF and tendon cells elongate due to tension [163]. In the abdomen, MTJ maturation follows a similar process but is complete only by 40 h APF [229].

#### 3.2.4. Sarcomere Assembly

Similar to embryos, premyofibrils are absent in DLM muscles. Mhc positive complexes are observed throughout the muscle by 26 h APF and assemble rapidly and synchronously across the entire muscle into myofibrils at 30 h APF immediately following tension generated by MTJ maturation [163,230]. This myofibril assembly fails in the absence of muscle attachment. The terminal Z-disc attaches to the MTJ mediated by integrins and IAPs [52]. Myofibrils are refined to regular arrays of sarcomeres over the next several hours where more sarcomeres are added. DLM myofibrils are flanked by MT arrays during initial stages of assembly that are dissembled by the end of pupation. The myofibril length then increases without other structural changes to reach its final length shortly after eclosion [231]. In the IFM, distinct transcriptional dynamics are associated with different stages of myofibrillogenesis, with the iTF Salm contributing to the transition after 30 h APF and its expression is maintained to establish IFM fate [230,232]. A similar sequence of myofibrillogenesis occurs in abdominal muscles that form mature MTJ by 50 h APF when myofibril assembly synchronously starts and is refined further to form the transversely aligned sarcomeres seen in abdominal muscles. Thin and thick filament complexes appear separately, then start interdigitating to form immature myofibrils by 46 h when muscles have stably attached to MTJ and exhibit spontaneous contractions. They subsequently assemble into ordered myofibrils by 50 h APF and are refined over the next several hours to begin coordinated contraction [229].

During IFM sarcomere assembly, thin filaments elongate from their pointed ends as is the case during embryonic myogenesis [170]. They initially form a dispersed pattern by the polymerization of actin into nascent thin filaments which become regularly patterned after 30 h APF. At this time, active incorporation of actin at both ends of the thin filament and further refinement and growth occurs by new actin monomer incorporation at the pointed ends of thin filaments and the formation of new thin filaments at the sarcomere periphery. Tmod and Sals that are located to pointed ends are necessary for thin filament length control [170,233]. The nebulin repeat containing protein Lasp regulates thin filament length by regulating its stability [234]. The *Drosophila* formin Fhos mediates thin filament assembly by initially regulating actin monomer incorporation into thin filaments during mid pupal stages and then localizes near Z-discs to facilitate radial growth of thin filament arrays to increase myofibril diameter [233]. In *Drosophila*, IFM thick filaments are associated with many insect-specific proteins such as myofilin [235], arthrin which is a ubiquitinated actin [236], paramyosin [237], minipramyosin [238], and flightin [239,240] not found in vertebrates, which could represent proteins adapted for flight [241]. The insect and IFM specific protein flightin is implicated in regulating the thick filament length by associating with myosin filaments as they grow [242,243]. Z-disc formation fails in the IFM if actin lacks its α-actinin binding domain showing the importance of sarcomere component interdigitation [244]. A downregulation of Sls results in smaller Z-discs around which a normal thick filament assembly occurs with abnormally long thick filaments at the periphery lacking the Z-disc [164,245]. As myofibrils grow, the Z-disc protein Zasp controls the final myofibril diameter by switching to growth restricting isoforms [246]. After complete myofibril growth, coordinated contractions can be initiated after mature NMJ formation.

#### 3.2.5. Innervation and NMJ Formation

Embryonic neuroblast lineages undergo a second larval wave of neurogenesis where embryonic neuroblasts are re-specified to give rise to adult MN lineages whose dendrites are organized in a ‘myotopic map’ within the CNS that reflects the innervation pattern of their target adult muscles similar to embryonic/larval stages [247,248,249,250]. MNs innervate adult muscles in a stereotypical pattern. For DLMs generated from larval templates, the primary larval ISN branch remains while secondary branches are initially retracted, and extensive new branching is generated as the muscles fuse with adult myoblasts and then split. Initial nerve arrival is muscle independent, but subsequent nerve branching occurs only in the presence of the target muscle [251]. Among DVMs, DVM I and DVM II are innervated by new branches arising from the larval ISN while the larval SN innervates DVM III [251,252]. The 14 leg muscles are innervated by around 50 MNs arising from specific neuroblasts in the CNS in a stereotypical pattern [250]. Following initial innervation, the NMJ is formed by extensive branching and synapse formation. The glial cells at the IFM NMJ express the glutamate *Drosophila* Excitatory Amino Acid Transporter 1 (dEAAT1) unlike during other stages for efficient neurotransmission [253]. Muscle iTFs contribute to correct innervation since malformed muscles cause MN branching aberrations as has been shown for Ewg [220].

In the end, a stereotypical muscle pattern along with stereotypical innervation generates fully functional adult muscles.

#### 3.2.6. Programmed Cell Death Following Eclosion of New Adults

Some larval abdominal muscles persist through metamorphosis and are used for the eclosion of new adults. These muscles degenerate after eclosion along with associated nerves [254].

## 4. The Maintenance of Muscle Homeostasis

### 4.1. Muscle Homeostasis under Normal Conditions

Functional larval somatic muscles and adult muscles represent two different homeostatic states during the fly lifetime. The embryonic wave of myogenesis takes only one day leading to the formation of functional larval muscles, which undergo continuous growth and refinement during the larval stages spanning five days. Larval muscle homeostasis needs to be coordinated with larval growth during the three larval instars until metamorphosis to ensure functional stability. Following metamorphosis and the pupal wave of myogenesis over a period of five days, adult flies eclose from their pupae and adult muscle homeostasis needs to be maintained during the fly lifespan of several weeks.

The stereotypical muscle pattern is associated with iTFs and their realisator genes that also exhibit tightly controlled spatial and temporal expression patterns in larval and adult muscles. Therefore, some of the iTFs can play a key role in the maintenance of muscle specific homeostasis by regulating the levels of key myogenic factors such as Mef2 as well as the expression of realisator genes [111,112,129,137] (Figure 2). The control of the level of activity of the key differentiation TF Mef2 is quintessential throughout the fly lifetime since this in turn controls the muscle specific levels of its vast array of target genes [118,129]. In the embryo, various genes were shown to require different Mef2 activity, with early expressing genes such as *Act57B* requiring lower levels compared to late expressing genes such as *Mhc* [118]. In the adult, the development and maintenance of the adult DLM muscles have been observed to be sensitive to the levels of Mef2 as well as its antagonist Holes in muscles (Him). Tubular adult muscles such as the TDT and DVM muscles seem to require lower Mef2 activity than the fibrillar DLM muscles since RNAi lines affect these muscles differently [255,256]. TFs such as Cf2 and E2f1 acting along with Mef2 could also contribute to setting the muscle homeostatic state [122,167]. A study identified putative Cf2 and Mef2 binding site clusters for multiple sarcomeric genes including *Mhc*, *Tm1*, *Tm2*, *up*, *wupA* (or *TnI*), and *paramyosin* (*Prm*) [122]. On Cf2 depletion, the stoichiometry of proteins such as TnT, TnI, and Prm was found to be altered and this imbalance worsened over the course of development. Another study detected E2f binding site enrichment upstream of myogenic genes such as *how*, *sals*, *Tm1*, *Mef2*, etc. This study also showed that E2f1 depletion altered the gene expression levels of *Tm2*, *Act88F*, *Mlc2*, *how*, and *Mef2* [167].

One hallmark of muscle homeostasis in *Drosophila* larval and adult muscles is the expression of fiber specific protein isoforms. Many sarcomeric genes switch between embryonic, larval, and/or adult isoforms during development, with different muscle types also exhibiting isoform specificity. Isoform switching usually occurs by switching to a predominant isoform. Embryonic *Mhc* transcripts contain exon 19, which is spliced out of adult versions and results in a different carboxy terminal [242,257]. Embryonic isoforms lack the functionality for the high ATPase rate and sliding velocity required for adult muscles [258]. The IFM muscles initially express an Mhc isoform containing exon 19 and switch to the adult exon 18 containing isoform during late stages of myofibril assembly [242]. A shorter embryonic/larval isoform of the pointed end capping protein Tmod is associated with actin during pupal sarcomere assembly and there is a switch to a longer Tmod isoform in eclosed adults [170]. Adult *Drosophila* muscles express fiber specific actins, with Act88F being expressed in the IFM and Act79B in the TDT, for example [202]. Two IFM specific Tm1 isoforms are expressed in adult flies [259,260]. Kettin is the predominant *Drosophila* titin isoform in embryos and the IFM muscles switch to the IFM specific predominant long Sls(700) isoform [245]. Zasp52 and other Zasp proteins also switch to adult isoforms [246,261], with Zasp52 expressing an exon 8 containing isoform absent in embryos, but present in the IFM and TDT. Obscurin expresses a single larval isoform and two IFM isoforms [262].

Isoform switches are potentially associated with cis regulatory modules (CRMs) that seem to be arranged in sequential modules mirroring developmental expression and regulation by different TFs and cofactors. Marin et al. identified an upstream regulatory element (URE) and an intronic regulatory element (IRE) in intron 1 of the *wupA* (or *TnI*) gene that acted synergistically and was capable of driving LacZ tagged TnI expression. Mas et al. identified similar elements in the *up* (or *TnT*) gene [263]. They showed that these elements synergistically interact in larval muscles, whereas the contribution of the IRE is higher in adult muscles. In addition, they showed that there was decreasing IRE contribution from the IFM to the jump muscles to the visceral muscles [264]. Garcia-Zaragoza et al. followed up on this study and identified the URE and potential IRE elements of *Tm1*, *Tm2*, and *Mhc*. *Tm1* was previously shown to be coordinately regulated by two intronic enhancers in cooperation with Mef2 and its interactor PAR domain protein 1 (Pdp1) [265,266,267]. Mature muscles need to ensure the activation and maintenance of the correct protein isoforms [268] since aberrant isoform expression impedes muscle function. For example, transient overexpression of a shorter Tmod isoform during mid-to late IFM assembly leads to normal length thin filaments at the periphery of the myofibrils that are correctly capped by the long Tmod isoform. However, they exhibit shorter core thin filaments within the myofibril caused by the permanent association of the shorter Tmod at their pointed ends, which cannot be dynamically uncapped to permit thin filament elongation. Therefore, this prevents its elongation causing defective sarcomeres that interfere with flight during adult stages [170]. The embryonic Mhc isoform fails to substitute for the IFM isoform due to different physiological properties [258,269].

Post transcriptional mechanisms such as phosphorylation could potentially contribute to muscle homeostasis. Thin and thick filament disruptions, for example, are associated with concomitant flightin phosphorylation deregulations [239]. Tm1 IFM isoforms are phosphorylated only in adult flies, which could have functional implications [260]. Impaired Talin phosphorylation leads to severe muscle detachment at late embryonic stages [154]. This means the right CRM regulatory mechanisms as well as post translational mechanisms such as phosphorylation [48,270] need to be dynamically maintained since specific protein domains are necessary for muscle specific functionality [269,271,272].

The accumulation of insoluble protein aggregates in the muscle is associated with protein aggregate myopathies (PAM) and in *Drosophila*, p38b deficiency leads to the deposition of polyubiquitinated protein aggregates in adult thoracic muscles and to locomotor defects [273]. Loss of components of the proteasome, which mediate protein turnover were shown to cause protein aggregates and progressive muscle atrophy in larval muscles [274]. Ubiquitin protein ligases such as Mind bomb 2 (Mib2) and Ubiquitin protein ligase E3A (Ube3A), which tag proteins for proteasomal degradation, have been associated with muscle defects. The loss of function of *mib2* was shown to trigger embryonic muscle apoptosis [275] and the over or under expression of *Ube3a* alters larval NMJ neurotransmission with associated altered number of active zones [276]. Proteostasis is thus integral to muscle maintenance.

**Figure 3:**
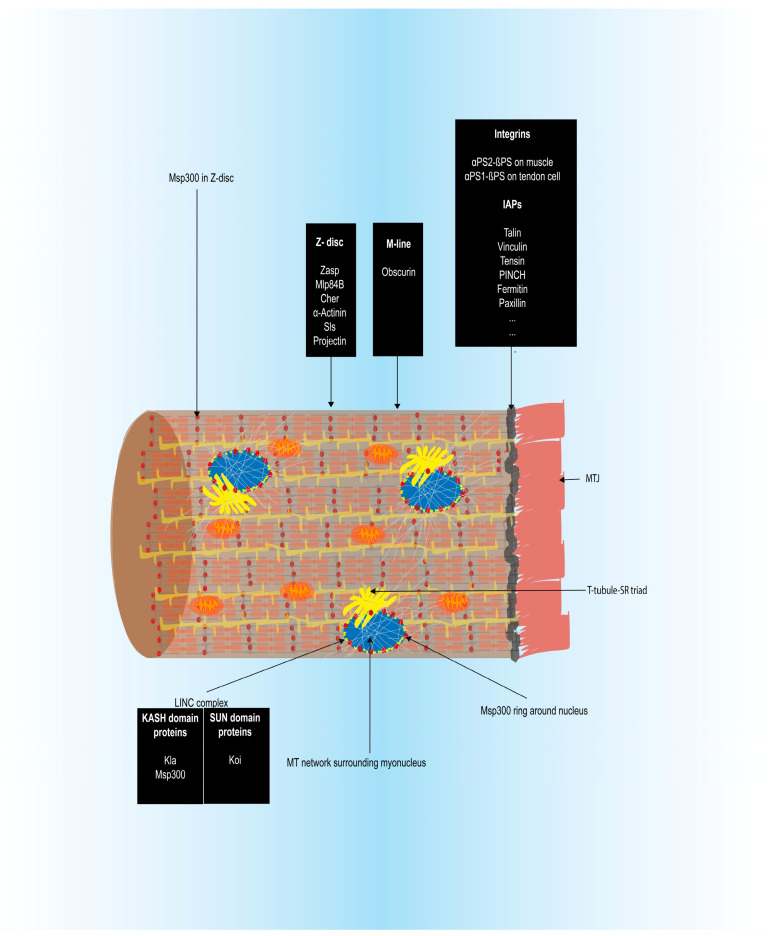
Maintenance of myofibril integrity and homeostasis. The integrin complex links the myofibrils to the MTJ via the extracellular matrix (ECM) and senses the forces transmitted by the MTJ. Integrins and Integrin Associated Proteins (IAPs) constitute the integrin complex. Integrin complex turnover and constitution are adapted to the forces sensed during contraction. The dense microtubule (MT) network anchored to the myonuclei by the Msp300 ring associated with the LINC complex on the nuclear envelope provides myonuclear elasticity during contractions to prevent disintegration of myonuclei and dissociation of the myofibril network. Msp300 in the Z-disc ensure regular spacing of organelles such as mitochondria and the SR for contractions. Z-disc and M-line components provide anchorage and elasticity to ensure sarcomeric integrity.

Muscle contraction is associated with multiple biochemical and morphological changes as well as large mechanical strains. This necessitates efficient mechanisms to withstand these forces to prevent muscle disintegration during contraction and to reinstate the stable muscle state (Figure 3). Protein stoichiometry is integral to sarcomere integrity since varying the expression levels of one protein has a cascading effect on the levels of other sarcomeric proteins leading to altered muscle functionality [277,278]. Sarcomeric integrity during contractions is maintained by components such as Mlp84B, Cher, small heat shock proteins (sHsps) such as dCryAB and Hsp67Bc and integrin-mediated adhesions. Mlp84B localizes to the Z-disc and genetically interacts with Sls. Mlp84B-Sls transheterozygotes exacerbate individual mutant phenotypes disrupting myofibrillar integrity [56]. Cher also interacts with Sls in addition to actin stably anchoring them to each other [53]. In addition, Cher interacts physically with dCryAB and a disruption of this interaction affects sarcomeric integrity [279]. The chaperone Hsp67Bc also colocalizes to the Z-disc although its function is unknown [280]. Integrin mediated adhesions maintain sarcomeric integrity and reduced adhesion results in the progressive age-dependent loss of sarcomeric cytoarchitecture [57]. Integrin and IAP stoichiometries at the MTJ are important to respond to different types of forces [166]. The myonuclear LINC complex and associated components such as Msp300 and Spectraplakin, which regulate MT organization, play a role in myonuclear maintenance by providing elasticity to resist contractile forces with the help of the MT network that surrounds it [281,282,283]. In addition, Msp300 associates with the Z-disc and keeps the mitochondria and SR anchored to the Z-disc during contractions [284]. Its presence around myonuclei near the larval NMJ also regulates glutamate receptor density to control locomotion [285].

NMJ activity perturbations lead to homeostatic synaptic plasticity, which enables compensatory modulations of the NMJ synaptic strength to resist these perturbations and stabilize synaptic activity. Lifelong synaptic plasticity ensures efficient neurotransmission of signals at the NMJ. The NMJ adapts various homeostatic mechanisms to maintain appropriate muscle function levels [286,287,288,289]. Mutants for *endophilin* (*endo*) exhibit tremendous synaptic overgrowth, but the overall synaptic strength is stabilized by reducing the active zone number in synaptic buttons, which modulates neurotransmitter release [286]. The NMJ adapts a homeostatic scaling mechanism called presynaptic homeostatic potentiation (PHP), where there is a compensatory increase in neurotransmitter release to maintain muscle excitation in response to abnormally reduced GluR on the postsynaptic end. This compensation appears to be associated with an uncharacteristic multilayer ring of electron dense T-bars in active zones to increase the neurotransmitter release [289]. The PHP maintenance has been shown to require inositol triphosphate (IP_3_) directed signaling [290]. During the larval growth spurt, NMJ homeostasis needs to be maintained even though the presynaptic end grows slower than the muscle surface that tends to accumulate GluRs. Ziegler et al. showed that the amino acid transporter, Juvenile hormone Inducible-21 (JhI-21) is a gene that coevolved with GluRs, is expressed at presynaptic ends and plays a role in suppressing excess GluR accumulation [288].

The close association of the mesoderm with other germ layers right from the embryonic stage and the continued association with epidermal and nervous tissues over the fly lifetime highlights the importance of coordinated intrinsic and extrinsic signaling for homeostasis.

### 4.2. Re-Establishment of Muscle Homeostasis Following Muscle Injury

Muscle regeneration has not been described in the larva. However, the larval stem cell-like AMPs that are capable of differentiating and giving rise to adult muscles were noted to have similarities to vertebrate muscle stem cells (MuSCs), also known as satellite cells. Similar to MuSCs, the Notch pathway [209,291] and zinc-finger homeodomain 1 (Zfh1), the *Drosophila* homolog of the vertebrate ZEB1/ZEB2 [292,293], maintain the AMPs in an undifferentiated state and they are capable of self-renewal by asymmetric divisions [209,294]. In addition, they are capable of fusion with existing larval muscle remnants during the formation of DLM muscles, which is reminiscent of muscle repair. It was initially thought that all muscle stem cell-like cells or AMPs are depleted during adult muscle formation and thus adult muscles were believed to lack regenerative capacity. Recently, Chaturvedi et al. identified a population of Zfh1 positive adult stem cells closely apposed to the adult muscle, which appear to possess the ability to proliferate and contribute to muscle regeneration upon injury [61] similar to vertebrate MuSCs [293]. Boukhatmi and Bray subsequently showed that Notch directly regulates Zfh1 to antagonize the differentiation of these cells by expressing a short Zfh1 isoform transcribed from an alternate promoter that is not subject to regulation by the conserved micro RNA, miR-8 [292]. Using the G-TRACE method for cell lineage analysis, their study showed that these cells, which they termed population of progenitors that persist in adults or pMPs, were mitotically active and incorporated into adult muscles even under normal conditions. Thus, they reiterated that these cells contributed to adult muscle homeostasis. Since this is a recent discovery, further studies could provide insights into the extent of repair in *Drosophila* adult muscles and the mechanisms involved in re-establishing and maintaining muscle identity and homeostasis.

### 4.3. Muscle Homeostasis under Pathological Conditions

Multiple studies in *Drosophila* models have reproduced defects observed in human pathological conditions and could provide important insights into disruptions of muscle homeostasis under pathological conditions. Many myopathies and neuromuscular disorders are associated with or even caused by myonuclear defects and others are associated with sarcomeric defects leading to muscle dysfunction, wasting, and/or degeneration. *Drosophila* models exist for multisystemic disorders such as Myotonic Dystrophy Type 1 (DM1) that is caused by CTG expansions in the *Dystrophia Myotonica Protein Kinase* (*DMPK*) gene leading to the sequestration of RNA binding proteins such as MBNL1 in nuclear foci [295,296]. This causes a disruption of muscle homeostasis as indicated by the progressive muscle degeneration observed in the IFM muscles in a model expressing 480 CTG repeats. The Dystrophin (Dys)-Dystroglycan (Dg) transmembrane complex at the plasma membrane acts as a crucial signaling mediator by relaying information to and from muscles to interacting tissues via the ECM. Mutations in genes constituting this complex or their interactors thus cause a disruption of homeostasis, thereby causing diseases such as Duchenne Muscular Dystrophy (DMD) where there is muscle wasting. In *Drosophila*, *Dg* was shown to be under miRNA regulation by miR-9a to ensure correct MTJ formation [297]. Large scale genetic and interactome screens in *Drosophila* have identified factors affecting muscle integrity such as stress response components [298] and components of the Hippo signaling pathway [42].

Laminopathies are disorders caused by mutations in the human *LMNA* genes which code for lamins present in the INM providing structural support and regulating gene expression. One *Drosophila* model revealed increased reductive stress due to the nuclear translocation of Nrf2, which is normally sequestered in the cytoplasm and released only during oxidative stress [299]. Chandran et al. observed a loss of muscle proteostasis in a *Drosophila* model of laminopathies and corroborated this by RNA-seq analyses of human muscle biopsy tissues. Interestingly, they were able to rescue the muscular phenotypes by the modulation of the AMPK pathway which could present future therapeutic directions [300]. Apart from laminopathies, other myopathies such as Centronuclear Myopathies (CNM) are associated with myonuclear positioning defects [301]. Muscle development studies in *Drosophila* are beginning to unveil mechanisms for myonuclear positioning and factors that disrupt this [141,284,302,303].

Other studies in *Drosophila* are providing insights into pathological features caused by disruptions in sarcomeric components. Muscular phenotypes caused by a mutation in the *Tm2* gene was found to be rescued by a suppressor mutation in the *wupA* gene coding for TnI [304]. *Drosophila* models exist for myosin myopathies such as Inclusion Body Myopathy Type 3 and Laing Distal Myopathy (LDM). A study has shown that the formation of large aggregates in muscles similar to those seen in human patients with *ZASP* mutations is caused by an imbalance in the levels of Zasp isoforms [246]. Dahl-Halvarsson et al. showed that the overexpression of the Thin protein, a homolog of the human TRIM family of proteins that is implicated in maintaining sarcomeric integrity, could alleviate LDM-like phenotypes [305].

## 5. Discussion

In vertebrates, the loss of skeletal muscle homeostasis is the cause of various muscular disorders. Studies in vertebrate systems are complicated by the presence of large gene families for multiple genes. *Drosophila* is a simple model organism with various conserved pathways and genes to study muscle homeostasis while at the same time mostly having one to a few genes orthologous to large vertebrate gene families that perform functions similar to vertebrate genes. Thus, it appears that genes are reused/repurposed over the course of evolution instead of ‘reinventing the wheel’. *Drosophila* muscle development has been studied for decades. The embryonic somatic muscles being uni-fiber muscles present a simple model to study development since all muscles have been well characterized along with their specific attachment sites and innervating MNs [143,180]. The IFM muscles have been equally well characterized [31,221,252]. In addition, a large number of tools are available in *Drosophila* to study in vivo mechanisms [306].

A better understanding of developmental and post developmental processes would help us gain a better understanding of the mechanisms of maintenance and disruption of homeostasis. The short life cycle of the fruit fly facilitates the quick and detailed study of processes making it a valuable model for the study of factors that initiate, maintain, and disrupt muscle homeostasis. The study of muscle regeneration following muscle injury, where developmental processes need to be re-initiated, represents an example of how the *Drosophila* model could help understand the mechanisms of muscle homeostasis. Some potential therapeutic targets have been unveiled by studies in *Drosophila* models of myopathies [300,305]. The recent discovery of stem cell-like cells associated with adult muscles is an exciting new direction of research to study muscle regeneration and homeostasis [61]. In vertebrates, aging is related to a depletion of the MuSC population leading to sarcopenia or age-related gradual loss of muscle mass and function [307] that is also characteristic of pathological conditions such as DMD [308]. The short life span of the *Drosophila* model presents a huge advantage to study homeostatic disruptions during aging.

Large gaps exist in our understanding of pathological mechanisms and simpler models could provide valuable insights and therapeutic directions. In *Drosophila*, although a lot of attention has been given to the major muscle components including the sarcomeres, MTJ and NMJ, muscle organelles that play an equally central role such as the SR, T-tubules, golgi complex, and transport vesicles have received lesser attention, although myonuclei are beginning to be studied in detail. Given the detailed characterization and tools available for this established model system that has already helped advance research [309,310], it would continue to serve as an important backbone for research into various physiological processes including muscle development and homeostasis.

## Figures and Tables

**Table 1:** The iTF expression patterns in embryonic somatic muscle founder cells.

iTF	Human Orthologs	FCs Expressing iTF ^1^	References	Embryonic Somatic Muscle Pattern
Apterous (Ap)	LHX	LT1, LT2, LT3, LT4, VA2, VA3	[78]	External muscles are represented in dark brown, intermediate muscles in a medium shade of brown, and internal muscles in fuchsia. 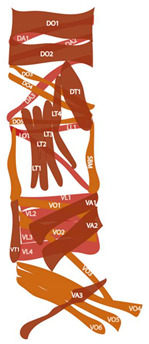
Araucan (Ara)	IRX	LT1, LT2, LT3, LT4, SBM , DT1- *DO3*	[87]	
Caupolican (Caup)	IRX	LT1, LT2, LT3, LT4, SBM , DT1- *DO3*	[87]	
Collier (Col)/Knot (Kn)	EBF	*DA2,*DA3-*DO5*,*DT1-DO3, LL1-DO4*	[82,85,88]	
Drop (Dr)/Muscle segment homeobox (Msh)	MSX	DO1, DO2, LT1-LT2, LT3-LT4, VA2, VA3	[89,90]	
Even-skipped (Eve)	EVX	DA1, *DO2*	[91,92]	
Krüppel (Kr)	KLF	DA1, DO1, *LT1*-LT2, *LT3*-LT4, LL1,*VA1*-VA2, *DO2*, VL3, VO2, VO5	[87,92,93]	
Ladybird (Lb)	LBX	SBM	[94]	
Lateral muscles scarcer (Lms)	-	LT1-LT2, LT3-LT4	[95]	
Midline (Mid)	TBX20	*LT3*-LT4, LO1, *VA1*-VA2	[96]	
Nautilus (Nau)	MYOD	DO1, DA2, DA3-DO5, DO3, LL1- DO4, LO1, VA1	[79,85,88,97]	
Optomotor-blind-related-1 (Org-1)	TBX1	LO1, VT1, SBM	[98]	
Pox meso (Poxm)	PAX	DT1-*DO3*, VA1-VA2, VA3	[99]	
Ptx1	PITX	Ventral muscles	[100]	
Runt		DO2, VA3, VO4	[92,101]	
Slouch (Slou)/S59	NKX1	DT1-*DO3*, *VA1*-VA2, VA3, VT1, *LO1*	[77,80,87]	
Scalloped (Sd)	TEF-1	*All FCs transiently*, maintained in VL1, VL2, VL3, VL4	[86]	
Vestigial (Vg)	VGLL	DA1-DA2, DA3, LL1, VL1, VL2, VL3, VL4	[86]	
Tailup (Tup)	ISL	DA1, DA2, DO1, DO2	[81]	
Eyes absent (Eya)		Differential temporal expression in multiple FCs	[85,102]	
Six4	SIX	Differential temporal expression in multiple FCs	[102,103]	
Sine occulis (So)	SIX	*DA2*, DA3-DO5, *LL1*-*DO4*	[85]	
No ocelli (Noc)	ZNF	DA3-DO5	[85]	
ETS-domain lacking (Edl)	-	*DA2*, DA3	[85]	

^1^ In the ‘FCs Expressing iTF’ column, each FC name is shown in the colour corresponding to the muscle it generates as depicted in the figure in the column on the extreme right. FCs known to be generated from an asymmetric division of the same progenitor cell are hyphenated. FCs with transient expression are shown in italics.

**Table 2:** The iTF expression patterns in myoblasts of adult muscles.

Adult iTF	Human Orthologs	Adult Myoblast Expression	Embryonic iTF Function ^1^	References	Adult Flight and Leg Muscle Pattern
Vestigial (Vg)	VGLL	IFM	DA1-DA2, DA3, LL1, VL1, VL2, VL3, VL4	[211]	Indirect flight muscles (IFM) are shown in shades of red and the direct flight muscles (DFM) in dark brown. Among the leg muscles, only the tergal depressor of trochanter (TDT) muscles are highlighted in olive green. Other leg muscles are in a light shade of green. 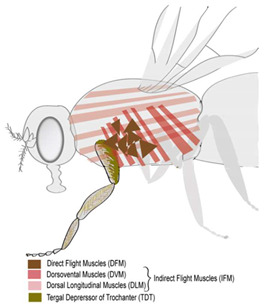
Extradenticle (Exd)	PBX	IFM		[218]	
Homeothorax (Hth)	MEIS	IFM		[218]	
Spalt major (Salm)	SALL	IFM		[219]	
Erect wing (Ewg)	NRF1	IFM		[220]	
Cut (Ct)		DFM		[213,214]	
Lateral muscles scarcer (Lms)	--	DFM	LT1-LT2, LT3-LT4	[95]	
Apterous (Ap)	LHX	DFM	LT1, LT2, LT3, LT4, VA2, VA3	[215]	
Ladybird (Lb)	LBX	Leg muscles	SBM	[214]	

^1^ In the ‘Adult myoblast expression’ column, names are shown in the colour corresponding to the muscles they generate as depicted in the figure in the column on the extreme right. Embryonic FCs known to be generated from an asymmetric division of the same progenitor cell are hyphenated.
